# Infantile Pompe disease with intrauterine onset: a case report and literature review

**DOI:** 10.1186/s13052-022-01379-3

**Published:** 2022-11-21

**Authors:** Hongmin Xi, Xianghong Li, Lili Ma, Xiangyun Yin, Ping Yang, Lulu Zhang

**Affiliations:** Neonatology Department, The Affiliated Hospital of Qingdao University, NO.16 Jiangsu Road, Shinan district, Qingdao, 266003 Shandong China

**Keywords:** Pompe disease, Glycogen storage disease type II, Acid maltase deficiency, Intrauterine onset

## Abstract

**Background:**

Pompe disease is a rare autosomal recessive disease. Acid alpha−glucosidase (GAA) deficiency leads to glycogen storage in lysosomes, causing skeletal, cardiac, and smooth muscle lesions. Pompe disease is progressive, and its severity depends on the age of onset. Classic infantile Pompe disease, the most severe form, is characterized by an age of onset before 12 months. Pompe disease with intrauterine onset has rarely been reported.

**Case presentation:**

The proband was born at a gestational age of 40 weeks and 3 days and admitted to our hospital because of intrauterine cardiac hypertrophy, shortness of breath, and cyanosis until 13 min postnatally. Physical examination at admission revealed poor responsiveness, pale skin, shortness of breath, reduced limb muscle tone, and bilateral pedal edema. The heart sounds were weak, and no heart murmur was heard. Echocardiography showed left (9 mm) and right (5 mm) ventricular hypertrophies. The patient was subjected to non−invasive ventilator−assisted respiration, fluid restriction, diuresis, and metoprolol treatment. Infantile Pompe disease was diagnosed on day 16 with a GAA enzymatic activity of 0.31 µmol/L/h and with the full−penetrance genetic test showing the homozygous gene mutation c.1844G>T(p.Gly615Val). Enzyme replacement therapy was refused by the patient’s parents, and the patient died at seven months of age from cardiopulmonary failure.

**Conclusion:**

Infants with intrauterine−onset Pompe disease usually have early manifestations of heart disease. Prompt GAA enzymatic activity determination and molecular genetic testing are helpful in aiding the parents’ decision and planning the treatment.

## Background

Pompe disease (MIM #232,300), or glycogen storage disease type II, is a rare autosomal recessive disease. Acid alpha-glucosidase (GAA) deficiency leads to glycogen storage in lysosomes, causing skeletal, cardiac, and smooth muscle lesions [1]. Pompe disease is progressive, and its severity depends on the age of onset. Classic infantile Pompe disease, the most severe form, is characterized by an age of onset before 12 months. Hypertrophic cardiomyopathy progresses rapidly with left ventricular outflow obstruction, generalized hypotonia, delayed motor development, feeding and swallowing difficulties, and dyspnea [2].

Pompe disease with intrauterine onset has rarely been reported, and the literature has not been reviewed. Obtaining a prenatal diagnosis is difficult because of the lack of specific manifestations. Herein, we report a case of infantile Pompe disease with intrauterine onset manifesting clinical symptoms postnatally, markedly reduced GAA enzymatic activity, and the presence of an uncommon homozygous mutation on genetic testing. Moreover, we review the literature and summarize the clinical characteristics and genotypes of reported patients with infantile Pompe disease with intrauterine onset to aid early diagnosis and treatment.

## Case presentation

A 40-week-3-day girl, weighing 4,090 g, was admitted to the hospital because of intrauterine myocardial hypertrophy for two weeks, shortness of breath, and cyanosis until 13 min postnatally. Electrocardiogram monitoring showed that her heart rate was 140 beats/min, respiration rate 60 beats/min and oxygen saturation was less than 90%.

**Fig. 1:**
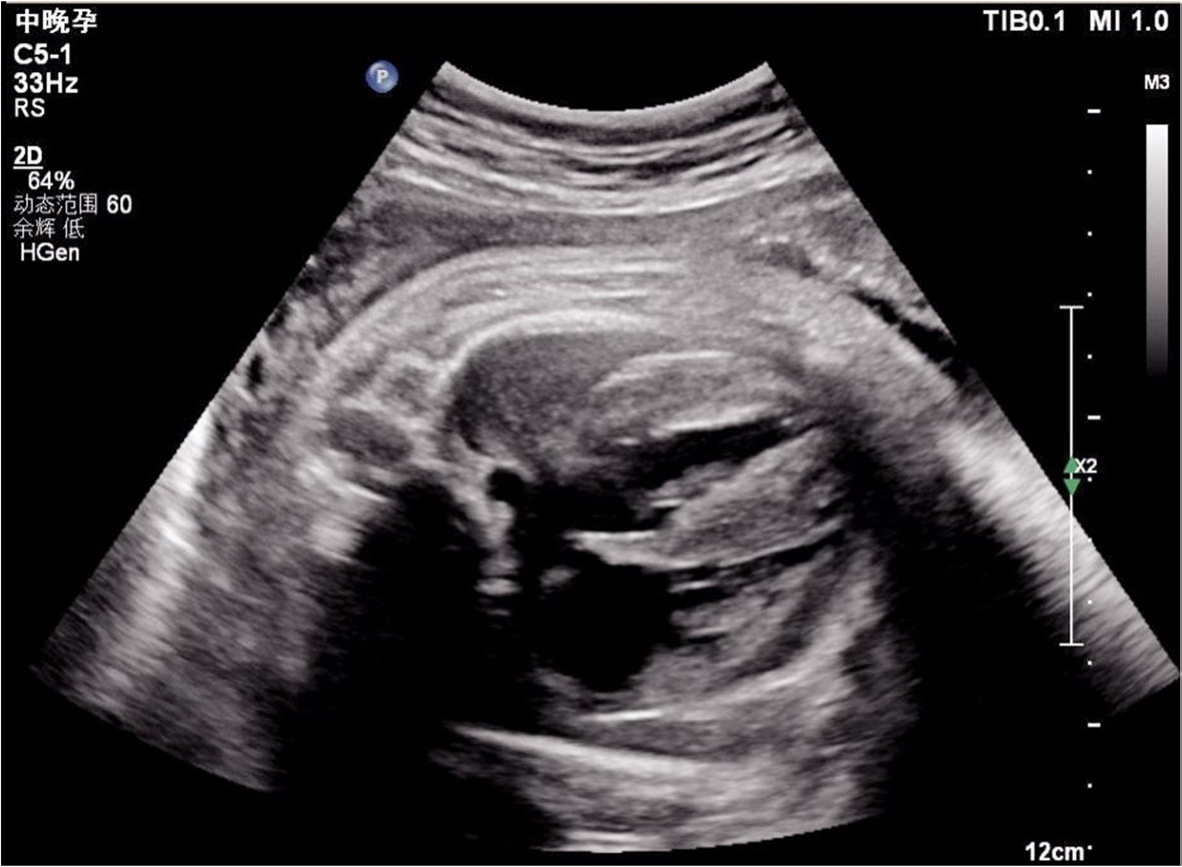
Echocardiogram demonstrating ventricular hypertrophy in utero

Physical examination on admission showed poor responsiveness, pale skin, irregular breathing, and positive Hoover’s sign without rales in both lungs. The heart sounds were low in intensity, but no heart murmur was heard. The liver was not enlarged. The limb muscle tone was reduced, and the lower limbs showed edema. The parents were healthy and non-consanguineous. At 38 weeks of gestation, ultrasound had shown an enlarged heart, with a significantly thickened left ventricular wall (interventricular septum, 10.4 mm; posterior left ventricle, 10.0 mm) and mild pericardial effusion (Fig. 1).

**Fig. 2:**
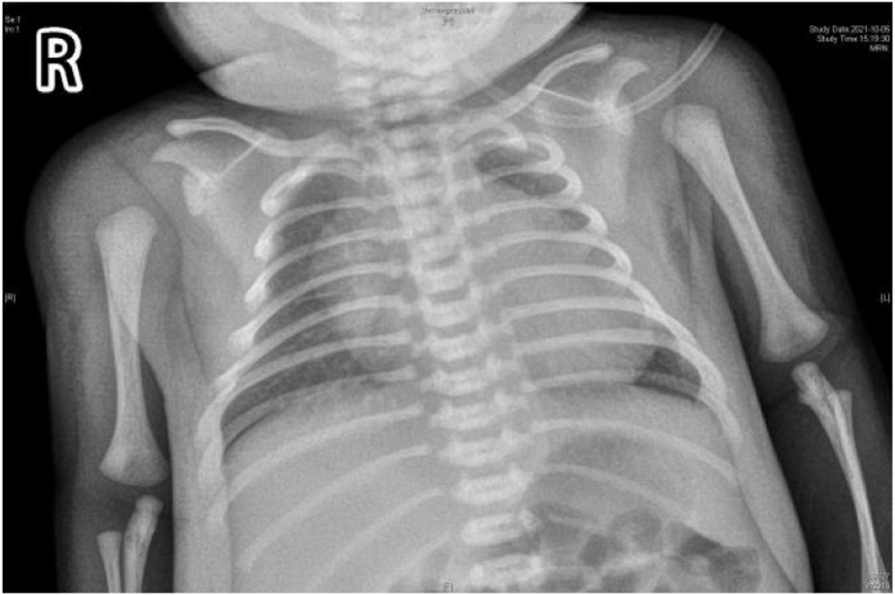
Chest films showing cardiomegaly with a cariothoracic ratio of approximately 80%

**Fig. 3:**
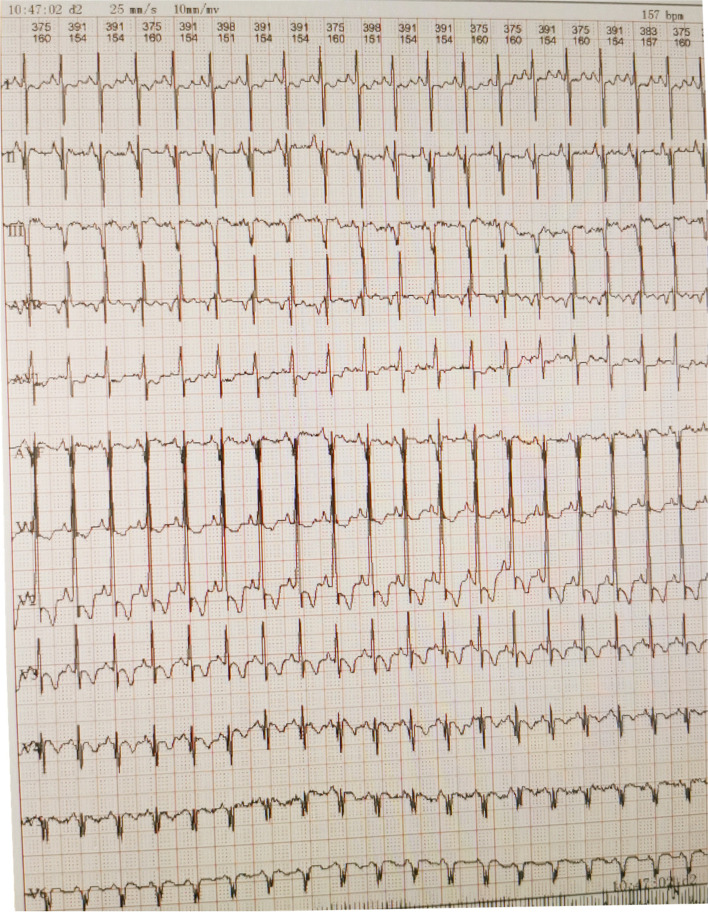
Electrocardiography revealing a short PR interval, high QRS voltage, ST-T changes, and a prominent Q wave

**Fig. 4:**
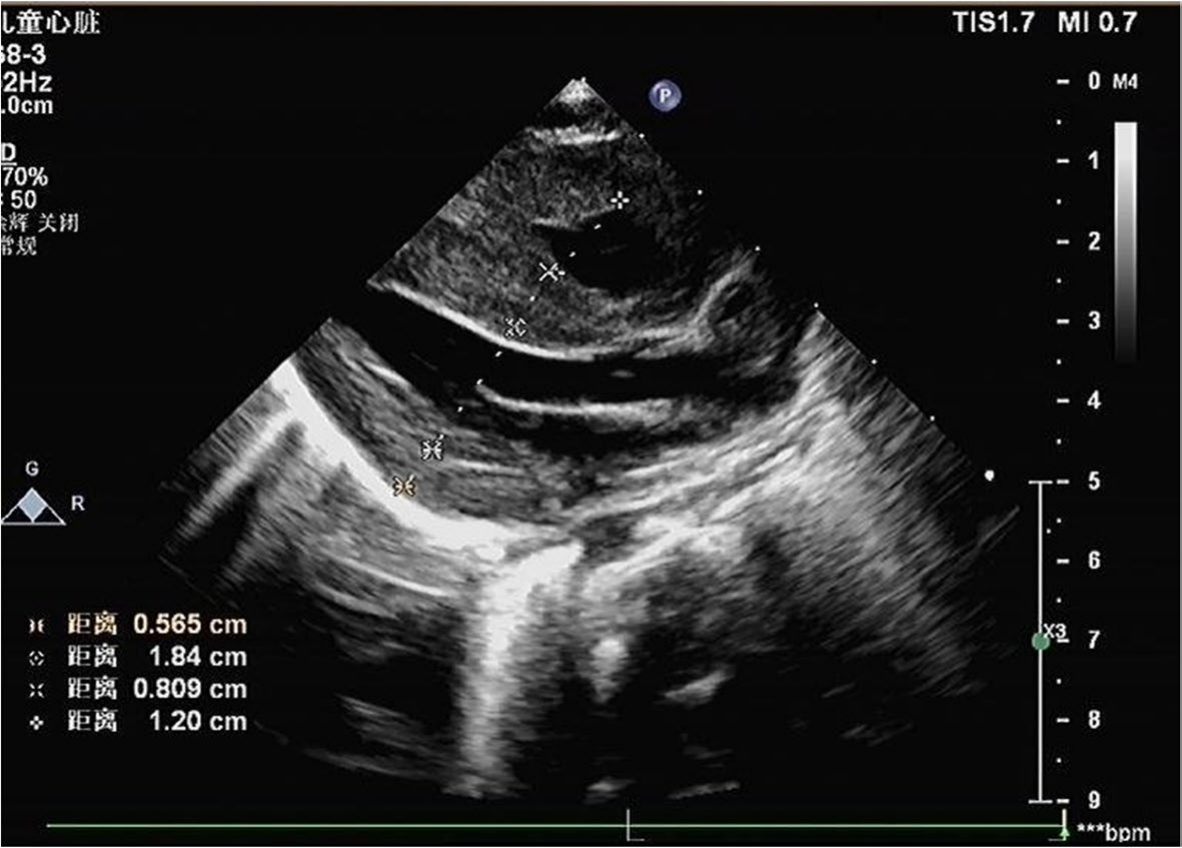
Echocardiogram demonstrating ventricular hypertrophy postnatally

**Fig. 5:**
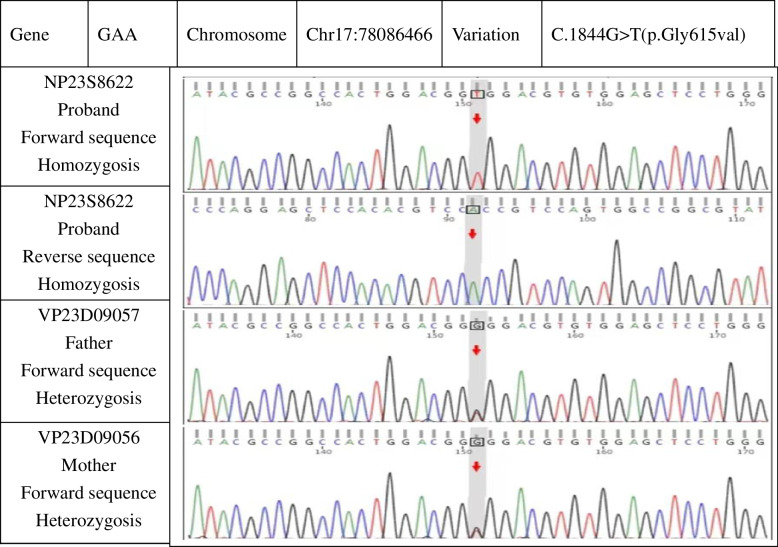
Genetic studies revealing a point mutation on exon 13 with Gly615Val and both parents carrying the recessive gene

After admission, laboratory findings included a B-type natriuretic peptide (BNP) level of 1,777.6 pg/mL, creatine kinase (CK) level of 3,594 ng/mL, and myoglobin level > 1,200.00 ng/mL. Chest X-ray showed a significantly increased cardiothoracic proportion (0.8; Fig. 2). The electrocardiogram showed a shortened PR interval, an abnormal Q wave, and ST-T changes (Fig. 3). Echocardiography showed heart enlargement, with significant thickening of the left ventricular wall (interventricular septum, 9 mm; Fig. 4). The patient was subjected to non-invasive ventilator-assisted breathing, nasal feeding, and metoprolol treatment to inhibit myocardial remodeling. Tandem mass spectrometry showed a GAA enzymatic activity of 0.31 µmol/L/h, and the full-penetrance genetic test showed the missense mutation c.1844G > T(p.Gly615Val). Subsequently, the patient was diagnosed with infantile Pompe disease (Fig. 5). The parents refused enzyme replacement therapy (ERT) because of financial constraints and the perceived poor prognosis.

**Fig. 6:**
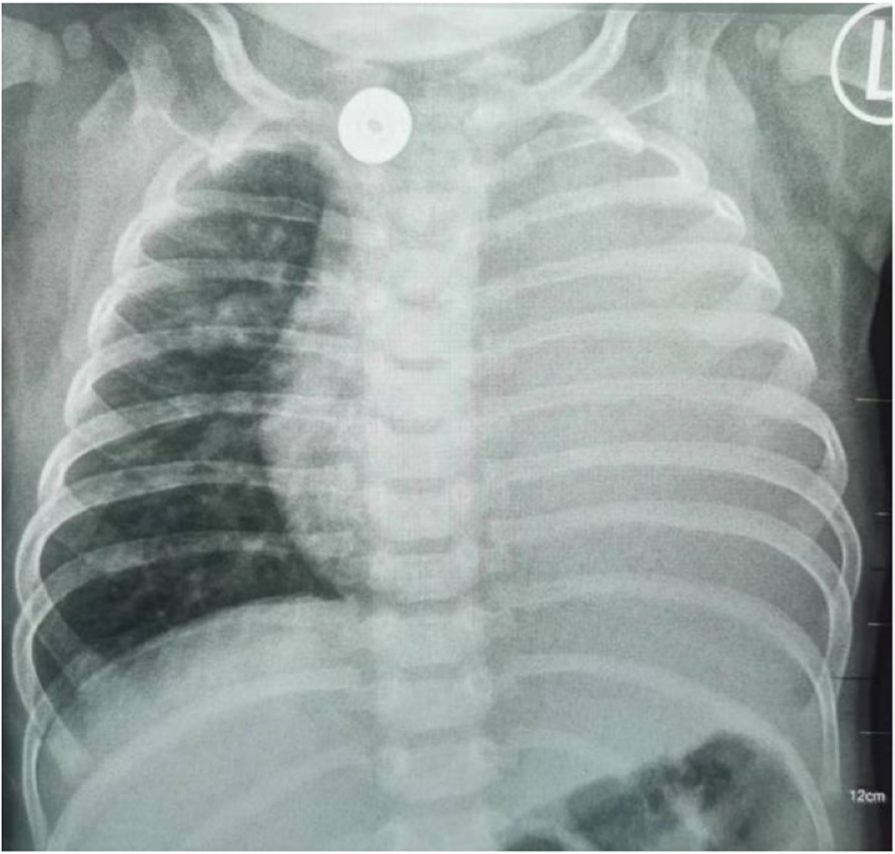
Patient showing left lung consolidation at 6 months of age

**Fig. 7:**
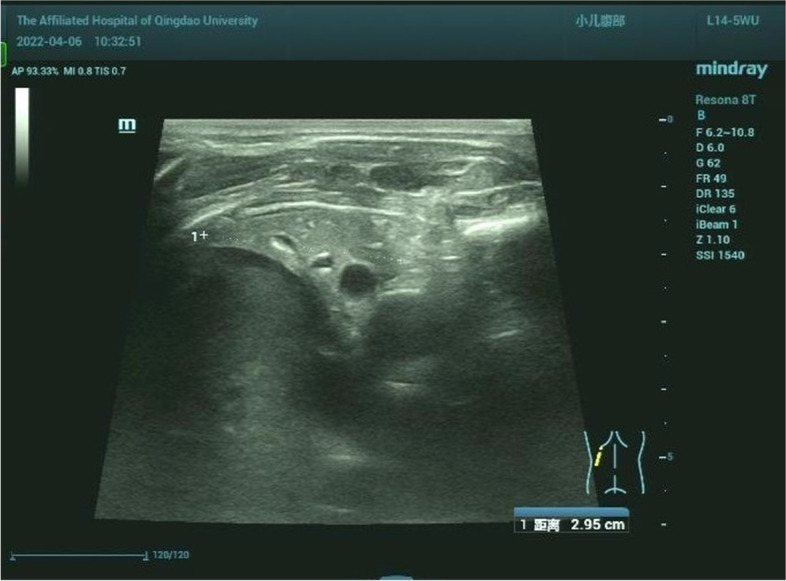
Left lung showing the same consolidation density as the liver

At six months of age, the patient weighed 7.5 kg and had a length of 70 cm. She could suck milk on her own and had no breathing difficulty. However, her crying was hoarse and weak, and muscle tension was significantly reduced throughout the body. She could not raise her head or turn over. Blood CK and BNP levels were 863.4 IU/L and 2,509.3 pg/mL, respectively. Chest X-ray showed left lung consolidation (Fig. 6). Chest ultrasound showed a left thoracic solid medium echo, consistent with lung tissue consolidation (Fig. 7). Echocardiography showed thickened ventricular septum and left and right ventricular free walls, measuring approximately 1.2, 1.0, and 0.65 cm, respectively. The left intraventricular and papillary muscle trabeculae were increased and thickened, and the ejection fraction was 57%. The patient died from cardiopulmonary failure at seven months of age.

## Discussion and conclusions

### Literature review

From inception to February 2022, we searched PubMed, Embase, Chinese National Knowledge Infrastructure, and WanFang databases using the following keywords: “Prenatal diagnosis” AND “Pompe disease” AND “Glycogen storage disease type II” AND “Acid maltase deficiency.” We extracted five English articles, reporting six cases of infantile Pompe disease with intrauterine onset [3–6]. Thus, including the present case, we reviewed seven cases (Table 1). The clinical characteristics of the patients were as follows:

**Table 1:** Characteristics and postnatal laboratory results of all patients

Patient No	Sex	GA/EFW at PE (weeks/kg)	GA at birth (weeks)	Laboratory results at birth (U/L)	GAA enzymatic activity (Assay site)	GAA mutations	Age at the first ERT	Survival time or FU duration	Respiratory support
1	M	32/2.7	35	AST,89 LDH, 457 CK,1199	Lymphocytes, 0.28 (normal: 4.8–13.3) μkat/kg	1327-2A > G (GAA inro 8)	18 h	Alive at 30 months	No
2	M	34/3.7	40	AST, 208 LDH, 994 CK,3685	Leukocytes, 3.7 (normal: 51–215) nmol/h/mg	1327-2A > G (GAA inro 8)	14 days	Died at 19 months	No
3	F	27/3.8	40	AST, 230 LDH, 1255 CK, 2381	Dried blood, 0.13 (normal: 7.3–39.0) pmol/punch/h	340insT (GAA ex 2–2)	2 h	Alive at 4 months	No
4	F	Not given	Full term	Not given	4 (normal: 24–94) pmoles/h/spot	c.1441 T > C (p.W481R) c.2481 + 109_c.2646 + 38del538	2 months	Alive at 14 months	Yes
5	F	31/3.50	38	BNP, 778 (pg/mL)	Not given	c.2560C > T(p. Arg854X)	2 days	Alive at 50 months	Yes
6	F	30/4.56	38	CK, 478	1.8 (control: 10–49) pmol/punch/h	c.525delT and c.1927 G > A (p.Gly643Arg)	None	Alive at 1.5 months	No
7	F	38/4.09	40	AST, 129 LDH, 1050 CK, 3594	0.31 (normal: 1.46–20.34) µmol/L/h	c.1844G > T(p.Gly615Val) GAA exon 13)	None	Died at 7 months	Yes

Normal range (U/L): AST, 15–40; LDH, 120–250; CK, 50–310

*GA* Gestational age, *EFW* Estimated fetal weight, *PE* Presentation, *AST* Aspartate aminotransferase, *CK* Creatine kinase, *BNP* B-type natriuretic peptide, *GAA* Acid α-glucosidase, *LDH* Lactate dehydrogenase, *ERT* Enzyme replacement therapy, FU Follow-up

### Sex

The patients included two boys and five girls.

### Age of onset and cardiomyopathy type

The gestational age of onset ranged from 27 to 38 weeks. Hypertrophic, dilated, and mixed (biventricular hypertrophy and left ventricular mass) cardiomyopathies were found in five patients (cases #1, #2, #3, #5, and #7), one patient (case #4), and one patient (case #6), respectively. The age at diagnosis ranged from antepartum to 6 months.

### Manifestations of cardiomyopathy

Five patients (cases #1, #2, #3, #5, and #7) showed left ventricular myocardial hypertrophy at onset. One patient (case #4) showed intrauterine dilated cardiomyopathy, which progressed to cardiac hypertrophy by two months of age. One patient (case #6) presented with biventricular hypertrophy with a left ventricular mass.

### Extracardiac manifestations

These patients showed no abnormality in appearance. Three patients (cases #4, #5, and #7) had dyspnea and decreased muscle tone postnatally and were admitted to the neonatal intensive care unit. The remaining four patients required no breathing support until the last follow-up.

### Myocardial enzymes

Levels of all myocardial enzymes were significantly elevated (478–3,685 U/L).

### GAA enzymatic activity determination and gene detection

The GAA enzymatic activity reduced significantly in six patients (case #6, data unavailable). Four patients had homozygous gene mutations: 1327-2A > G in two patients (cases #1 and #2), 340insT in one patient (case #3), and c.2560C > T(p. Arg854X) in one patient (case #6). Our patient (case #7) had the Gly615val(13) homozygous gene mutation. Two patients had compound heterozygous gene mutations: c.1441 T > c(p.W481R) and c.2481 + 109_c.2646 + 38del538 in one patient (case #4) and c.525delT and c.1927G > A(p.Gly643Arg) in the other patient (case #5).

### Outcomes

Five patients (cases #1, #2, #3, #4, and #5) received ERT, and myocardial hypertrophy improved notably. However, one patient (case #2) died, and one patient (case #4) experienced respiratory failure at 14 months and underwent tracheotomy. Of the two patients not treated with ERT, one (case #6) showed decreased muscle tone at six weeks, and the other’s (case #7) condition worsened, with severe hypotonia, progressive myocardial hypertrophy, decreased cardiac function, and left lung consolidation at six months, followed by death from cardiopulmonary failure at seven months.

## Discussion

Classic infantile Pompe disease has a median age of onset of 2.4 (range: 0.0–12.0) months and can manifest clinical symptoms at any age [7]. Intrauterine onset of Pompe disease is extremely rare. Combined with the present case, only seven cases of Pompe disease with intrauterine onset have been reported. Despite the term “infantile” in its name, the onset occurs before birth. Pompe disease has various manifestations. Infantile cardiomyopathy mainly manifests as left ventricular myocardial hypertrophy [8]. All seven neonates showed myocardial changes on intrauterine ultrasound, but manifestations varied, including hypertrophic cardiomyopathy, dilated cardiomyopathy, and a myocardial mass. All cases eventually progressed to myocardial hypertrophy postnatally. Levels of other laboratory indicators, such as myocardial enzymes and BNP, increased to varying degrees.

Neonatal hypertrophic cardiomyopathy and hypotonia should be distinguished from other diseases, such as glycogen storage disease type IIB (Danon disease), specific fatty acid oxidation disorder, and mitochondrial respiratory chain dysfunction. These diseases have similar clinical symptoms and are mainly diagnosed based on genetic testing results [9–11].

All patients had GAA-related gene mutations. Although the onset was intrauterine, prenatal diagnosis using a *GAA* mutation analysis of the amniocentesis sample could only be obtained in one patient (case #5), owing to the positive family history. Chien et al. suggested that the treatment prognosis is better when started in the first few days of life; therefore, early diagnosis is particularly important [12]. Prenatal diagnosis could be obtained based on the laboratory finding of GAA deficiency in amniotic fluid cultures or the molecular finding of *GAA* mutations [13]. Pompe disease is recessively inherited, and its genetic diagnosis using chorionic villus samples can only be made if the parents are known carriers. Contamination with maternal tissue when biochemical or molecular methods are used for the diagnosis yields a false-positive result [14]. Therefore, a targeted mutational analysis should be performed to confirm the prenatal diagnosis of Pompe disease [15]. Prenatal diagnosis could not be obtained in the present case because of the unknown family history.

Currently, ERT is the cornerstone of Pompe disease management [16]. ERT can improve left ventricular function and yield better outcomes after early ERT [17]. Therefore, newborn screening for Pompe disease is of great importance [18].

We reviewed seven patients, five of whom received ERT. The time of treatment onset ranged from two hours (case #3) to two months (case #4) postnatally. Two patients did not receive treatment (cases #6 and #7). The myocardial thickness returned to normal in all patients treated with ERT, but one patient (case #2) died from respiratory failure because of infection.

Our patient had homozygous gene mutations at the same site and presented with clinical symptoms postnatally. After symptomatic treatment, vital signs stabilized for a short time, but the patient had serious developmental delay. Further, the left lung developed consolidation, and myocardial hypertrophy increased with decreasing cardiac function and severe hypotonia at six months. The mechanism of respiratory failure in patients with Pompe disease mostly involves extensive pathological changes in the muscle and nerve components of the respiratory system resulting from glycogen accumulation [19]. However, no reported patient with Pompe disease has shown homogeneous solid changes in the lungs. A completely enzyme-deficient Pompe disease-knockout mouse model showed glycogen storage in nearly all tissue and cell types [20]. Another mouse model of Pompe disease showed lysosomal glycogen accumulation in tracheal and bronchial smooth muscles [21]. Therefore, lung consolidation in patients with primary disease is considered to be related to glycogen accumulation and deposition.

Although clinical symptoms of some patients treated with ERT improved, ERT had several limitations, including the risk of immunogenicity-related complications, inability to penetrate central nervous system tissue, and requirement of life-long therapy. Further, no trial compared the effectiveness or safety of ERT with that of other interventions or placebo until 2016 [22]. Gene therapy seems promising for potential prevention, halting, and reversal of Pompe disease [19]. However, only animal experiments have been conducted, and clinical studies had a small sample size, hindering the applicability of gene therapy [1]. None of the patients we reviewed received gene therapy.

Infantile Pompe disease is a rare hereditary disease that can occur in utero, with initial signs being cardiac changes. Therefore, for fetuses with intrauterine cardiac disease, enzyme assays and genetic testing should be performed as soon as possible for early diagnosis and treatment.

This study has some limitations. First, the number of cases was small. Second, the follow-up of some patients had not been completed at the time of reporting.

## Data Availability

The datasets generated and analyzed during the current study are shown in the manuscript.

## Abbreviations

IOPD: Infantile Pompe diseasel
GAA: Acid alpha-glucosidase
BNP: B-type natriuretic peptide
CK: Creatine kinase
HCM: Hypertrophic cardiomyopathy
DCM: Dilated cardiomyopathy
ERT: Enzyme replacement therapy

## References

[1] Meena NK, Raben N. Pompe Disease: New Developments in an Old Lysosomal Storage Disorder. Biomolecules. 2020;10(9):1339. Published 2020 Sep 18. doi:10.3390/biom10091339 10.3390/biom10091339 PMC7564159 32962155

[2] Llerena Junior JC, Nascimento OJ, Oliveira AS (2016). Guidelines for the diagnosis, treatment and clinical monitoring of patients with juvenile and adult Pompe disease. Arq Neuropsiquiatr.

[3] Hamdan MA, El-Zoabi BA, Begam MA, Mirghani HM, Almalik MH. Antenatal diagnosis of pompe disease by fetal echocardiography impact on outcome after early initiation of enzyme replacement therapy. J Inherit Metab Dis. 2010,33 Suppl 3S333-S339. 10.1007/s10545-010-9179-2. 10.1007/s10545-010-9179-2 20821053

[4] Tsai AC, Hung YW, Harding C, Koeller DM, Wang J, Wong LC (2017). Next generation deep sequencing corrects diagnostic pitfalls of traditional molecular approach in a patient with prenatal onset of Pompe disease. Am J Med Genet A.

[5] Swarr DT, Kaufman B, Fogel MA, Finkel R, Ganesh J (2012). Unusual cardiac “masses“ in a newborn with infantile pompe disease. JIMD Rep.

[6] Gupta P, Shayota BJ, Desai AK, et al. A Race Against Time-Changing the Natural History of CRIM Negative Infantile Pompe Disease. Front Immunol. 2020;11:1929. Published 2020 Sep 4. 10.3389/fimmu.2020.01929. 10.3389/fimmu.2020.01929 PMC7498628 33013846

[7] Reuser AJJ, van der Ploeg AT, Chien YH (2019). GAA variants and phenotypes among 1,079 patients with Pompe disease Data from the Pompe Registry. Hum Mutat.

[8] Van der Ploeg AT, Reuser AJ (2008). Pompe's disease. Lancet.

[9] Gu J, Geng M, Qi M, Wang L, Zhang Y, Gao J. The role of lysosomal membrane proteins in glucose and lipid metabolism. FASEB J. 2021;35(10):e21848. 10.1096/fj.202002602R. 10.1096/fj.202002602R 34582051

[10] Marsden D, Bedrosian CL, Vockley J (2021). Impact of newborn screening on the reported incidence and clinical outcomes associated with medium- and long-chain fatty acid oxidation disorders. Genet Med.

[11] Parikh S, Goldstein A, Koenig MK (2015). Diagnosis and management of mitochondrial disease: a consensus statement from the Mitochondrial Medicine Society. Genet Med.

[12] Chien YH, Hwu WL, Lee NC (2013). Pompe disease: early diagnosis and early treatment make a difference. Pediatr Neonatol.

[13] Bembi B, Cerini E, Danesino C (2008). Diagnosis of glycogenosis type II. Neurology.

[14] Fowler DJ, Anderson G, Vellodi A, Malone M, Sebire NJ (2007). Electron microscopy of chorionic villus samples for prenatal diagnosis of lysosomal storage disorders. Ultrastruct Pathol.

[15] van der Ploeg AT, Kruijshaar ME, Toscano A, et al. European consensus for starting and stopping enzyme replacement therapy in adult patients with Pompe disease: a 10-year experience. Eur J Neurol. 2017;24(6):768–e31. 10.1111/ene.13285. 10.1111/ene.13285 28477382

[16] Davison JE (2020). Advances in diagnosis and management of Pompe disease. J Mother Child.

[17] Yang CF, Yang CC, Liao HC, et al. Very early treatment for infantile-onset Pompe disease contributes to better outcomes. J Pediatr. 2016;169:174–80.e1. 10.1016/j.jpeds.2015.10.078. 10.1016/j.jpeds.2015.10.078 26685070

[18] Chien YH, Chiang SC, Zhang XK, et al. Early detection of Pompe disease by newborn screening is feasible: results from the Taiwan screening program. Pediatrics. 2008;122(1):e39–45. 10.1542/peds.2007-2222. 10.1542/peds.2007-2222 18519449

[19] Fusco AF, McCall AL, Dhindsa JS (2020). The Respiratory Phenotype of Pompe Disease Mouse Models. Int J Mol Sci.

[20] van der Ploeg AT, Reuser AJ (2008). Pompe's disease. Lancet.

[21] Keeler AM, Liu D, Zieger M, et al. Airway smooth muscle dysfunction in Pompe (Gaa-/- ) mice. Am J Physiol Lung Cell Mol Physiol. 2017;312(6):L873–81. 10.1152/ajplung.00568.2016. 10.1152/ajplung.00568.2016 PMC5495946 28336814

[22] Chen M, Zhang L, Quan S (2017). Enzyme replacement therapy for infantile-onset Pompe disease. Cochrane Database Syst Rev.

